# Editorial: Women in science - pulmonary medicine 2023

**DOI:** 10.3389/fmed.2024.1486414

**Published:** 2024-09-19

**Authors:** Tao Zhu, Zhihong Chen

**Affiliations:** ^1^Department of Respiratory Medicine and Critical Care Medicine, Suining Central Hospital, Suining, Sichuan, China; ^2^Department of Respiratory Medicine and Critical Care Medicine, Zhongshan Hospital of Fudan University, Shanghai Institute of Respiratory Disease, Shanghai, China

**Keywords:** women, pulmonary medicine, COPD, asthma, interstitial lung disease

Although the proportion of women in science, technology, engineering, and mathematics (STEM) has been gradually rising in recent decades, women are still under-represented both as scientists and as participants in medicine research. Globally, it is estimated that only 30% of researchers are women (1, 2). Meanwhile, evidence indicates that a sex bias still exists throughout many academic fields. In spite of this, women contribute substantially to advancing medicine knowledge (1, 2). Pulmonary diseases are one of leading causes of morbidity and mortality worldwide. The Research Topic “*Women in science - pulmonary medicine 2023*” highlights work led by women in the field of pulmonary medicine.

All manuscripts in this Research Topic have been undergone a rigorous peer review process. Ultimately, 10 publications (eight research studies, one clinical study protocol, and one review paper), which were all led by senior and early career women from across the world, focusing on lung health, were included.

COPD is one of leading cause of death. In this Research Topic, three research studies and one study protocol focused on different aspects of COPD management. Mou et al., investigated the anxiety-associated clinical profile in older patients with COPD. In this study, a total of 424 older COPD patients were enrolled. Among them, 84 had anxiety, and 340 were without anxiety. There data showed that the BODE index, mMRC, CAT score, comorbidities, and acute exacerbations were independently associated with anxiety in older COPD patients. Then, these results imply that anxiety accounts for worsening symptoms, more comorbidities, and frequent acute exacerbation in older patients with COPD. Thus, more attention should be provided to anxiety in COPD management. In a cross-sectional study, Shen et al. explored the values of five methods, COPD-PS scale, COPD-SQ scale, peak expiratory flow (PEF), COPD-PS scale combined with PEF, and COPD-SQ scale combined with PEF, in diagnosing COPD. They revealed that the sensitivity and specificity of both COPD-SQ questionnaire and COPD-PS questionnaire in prediction COPD was markedly promoted by combing with PEF. Furthermore, among them, the performance of COPD-SQ questionnaire combined with PEF was better. Meanwhile, Shi et al. designed a supervised, single-blinded, randomized controlled clinical trial to investigate the efficacy and safety of music-therapy facilitated pulmonary telerehabilitation program in COPD management. Then, shuttle walking test was used as primary outcome. Additionally, in a pre-clinical study, Brito et al. reported the role of photobiomodulation (PBM) in mediating cytokines, chemokines, and transcription factors expressions in variety of effector and regulatory T cells, such as CD4+STAT4 cells, CD4+CD25+Foxp3+ cells, etc, in the lung in a murine model of COPD. The results showed that lung pathological alterations, airway inflammation, and inflammatory mediators were attenuated by PBM, possibly through promoting regulatory T cells (CD4+CD25+Foxp3+) population and enhancing their IL-10 releasing in COPD mice.

Asthma is a common non-communicable airway disorder, affecting more than 300 million people worldwide (3). Severity evaluation is essential for asthma treatment. Mao et al. explored the values of neutrophils and related oxidative stress-associated molecules in peripheral blood and induced sputum in the prediction of asthma severity. Compared to non-severe asthma, severe asthma had higher levels of neutrophils, neutrophils%, and 8-iso-PGF2a in the peripheral blood, and increased ROS concentration of neutrophils in the induced sputum. Additionally, they also found that neutrophils and 8-iso-PGF2a in peripheral blood were the promising biomarkers for asthma severity prediction.

It is well-known that smoking is a common risk factor for a variety of diseases, particularly respiratory and cardiovascular system. Batista A. N. R. et al. explored the association between cardiac morphometric features and myocardial fat deposition in young adults. Meanwhile, the role of smoking cessation on the lipid metabolism were also investigated. In this cross-sectional study, it is found that smoking history was positively correlated with myocardial triglyceride (TG) deposition. Then, compared to non-smoking group, high-density lipoprotein cholesterol was lower, and TG and very-low-density lipoprotein cholesterol were higher in smoking group. Their findings indicate smoking can lead to cardiac morphometric abnormal and promote myocardial fat deposition. Then, smoking cessation improves cardiac function and lipid profile disorder. Otherwise, an exciting review was presented by Batista D. R. et al. about the relation of electronic nicotine delivery systems (ENDS) exposure and metals in biological samples. It was showed that both primary and second-hand electronic nicotine delivery systems (ENDS) exposure resulted in increased levels of a variety of metals, including lead and cadmium, in biological samples. Meanwhile, they also revealed that conventional combustible cigarette users have similar or higher metal levels than ENDS users.

Interstitial lung disease (ILD) is a group of pulmonary disorders characterized by inflammation and/or fibrosis (4). Idiopathic pulmonary fibrosis (IPF) accounts for approximately more than 30% of all cases of ILD. However, its etiology and pathogenesis are still not clear. Liu et al. explored the potential key genes in IPF and their roles in immune cells using integrated bioinformatics analysis. Subsequently, these findings were verified both *in vivo* and *in vitro*. They identified that CFH and FHL2 were essential for IPF, which also played the hub roles in different immune cells. Collectively, these results indicate that CFH and FHL2 were promising biomarkers for IPF diagnosis. Silicosis is another pulmonary fibrosis disease with poor prognosis. In a retrospective study, 246 adult patients with silicosis were included from China. Kang et al. used a novel inflammatory biomarker, the systemic immune-inflammation Index (SII), to assess the severity of silicosis. Then, they found that SII level was independently associated with advanced stage of silicosis. Additionally, 444.1 could be used as the cut-off value of SII index to predict advanced stage of silicosis.

Lastly, Fischer et al. developed a new questionnaire, the HeLP (Health Literacy in Pulmonary Embolism)-Questionnaire, which was composed of 23 items in four domains, to assess pulmonary embolism-specific issues of health literacy and to evaluate its psychometric properties.

Collectively, this Research Topic highlighted that current women-led investigations contribute substantially to pulmonary medicine. Then, we hope that it can further promote and inspire female medical researchers and clinicians to continue their explorations into novel advances in academic fields.
